# Changes in Growth, Photosynthesis Performance, Pigments, and Toxin Contents of Bloom-Forming Cyanobacteria after Exposure to Macroalgal Allelochemicals

**DOI:** 10.3390/toxins13080589

**Published:** 2021-08-23

**Authors:** Gracjana Budzałek, Sylwia Śliwińska-Wilczewska, Marek Klin, Kinga Wiśniewska, Adam Latała, Józef Maria Wiktor

**Affiliations:** 1 Division of Marine Ecosystems Functioning, Institute of Oceanography, University of Gdańsk, Piłsudskiego 46, P-81-378 Gdynia, Poland; gbudzalek@gmail.com (G.B.); marek.klin@ug.edu.pl (M.K.); adam.latala@ug.edu.pl (A.L.); 2 Division of Marine Chemistry and Environmental Protection, Institute of Oceanography, University of Gdańsk, Piłsudskiego 46, P-81-378 Gdynia, Poland; kinga.wisniewska@phdstud.ug.edu.pl; 3 Department of Marine Ecology, Institute of Oceanology of the Polish Academy of Sciences, Powstańców Warszawy 55, P-81-712 Sopot, Poland; wiktor@iopan.pl

**Keywords:** allelopathy, green algae, macroalgae, secondary metabolites, toxic cyanobacteria

## Abstract

Macroalgae can directly restrict the growth of various phytoplankton species by releasing allelopathic compounds; therefore, considerable attention should be paid to the allelopathic potential of these organisms against harmful and bloom-forming cyanobacteria. The main aim of this study was to demonstrate for the first time the allelopathic activity of *Ulva intestinalis* on the growth, the fluorescence parameters: the maximum PSII quantum efficiency (*F*_v_/*F*_m_) and the effective quantum yield of PSII photochemistry (ΦPSII), the chlorophyll *a* (Chl *a*) and carotenoid (Car) content, and the microcystin-LR (MC-LR) and phenol content of three bloom-forming cyanobacteria, *Aphanizomenon* sp., *Nodularia spumigena*, and *Nostoc* sp. We found both negative and positive allelopathic effects of *U. intestinalis* on tested cyanobacteria. The study clearly showed that the addition of the filtrate of *U. intestinalis* significantly inhibited growth, decreased pigment content and *F*_v_/*F*_m_ and ΦPSII values of *N. spumigena* and *Nostoc* sp., and stimulated *Aphanizomenon* sp. The addition of different concentrations of aqueous extract also stimulated the cyanobacterial growth. It was also shown that the addition of extract obtained from *U. intestinalis* caused a significant decrease in the MC-LR content in *Nostoc* sp. cells. Moreover, it the phenol content in *N. spumigena* cells was increased. On the other hand, the cell-specific phenol content for *Aphanizomenon* sp. decreased due to the addition of the filtrate. In this work, we demonstrated that the allelopathic effect of *U. intestinalis* depends on the target species’ identity as well as the type of allelopathic method used. The study of the allelopathic Baltic macroalgae may help to identify their possible role as a significant biological factor influencing harmful cyanobacterial blooms in brackish ecosystems.

## 1. Introduction

Macroalgal allelopathy is considered to be a special strategy to deter or eliminate competitors and predators coexisting in a shared ecosystem [1]. The secondary metabolites produced and secreted into the environment by donor organisms have been called allelopathic compounds [2]. Macroalgal allelopathy depends on the production and secretion of active allelopathic compounds and their efficient dispersal to target organisms that were affected by these compounds [3]. These compounds can not only affect other plants, but also cyanobacteria, protists, zooplankton, bivalves, crustaceans, and fish by limiting their reproduction and causing high mortality [4,5]. Allelopathic compounds can also affect the growth and photosynthesis of coexisting cyanobacteria and microalgae (e.g., [6,7,8,9]). However, many of the mechanisms of action of allelopathic compounds are not fully understood [10]. 

Allelopathy in aquatic ecosystems occurs in coastal areas where macroalgae and macrophytes are often present [11,12,13,14,15,16,17]. Macroalgae in the aquatic environment belong to green (Chlorophyta and Charophyta), red (Rhodophyta), and brown (Ochrophyta) algae. Macroalgae have been found to produce active metabolites that inhibit other organisms that compete with them for nutrients, light or space [18,19]. Thus, the competition for resources may be crucial in the occurrence of allelopathic interactions. Furthermore, the bottom in the littoral zone is limited compared to the pelagic offshore; thus, allelopathic interactions in coastal ecosystems may be stronger [20]. Consequently, allelopathic interactions may contribute to changes in phytoplankton and phytobenthic structures in aquatic ecosystems, especially in shallow waters [20].

Some studies have shown that compounds secreted by green macroalgae *Cladophora glomerata* inhibited the growth of the epiphytic diatom *Nitzschia* sp. [21]. In contrast, Anthoni et al. [22] suggested that the low abundance of epiphytes on *Chara globularis* thallus indicated that these macroalgae were probably also capable of producing harmful, unidentified allelopathic compounds. Seven years later, Anthoni et al. [23] isolated a compound called charamin, extracted from this species, which exhibited strong antibiotic activity. Two sulfur compounds (dithiolate and trithiane) isolated from *C*. *globularis* were also shown to inhibit the growth of the diatom *Nitzschia palea* and to reduce the abundance of natural phytoplankton communities [24,25]. In contrast, Mulderij et al. [26] studied the allelopathic activity of two species of *C*. *globularis* and *Chara contraria* on three species of green microalgae: *Selenastrum capricornutum*, *Chlorella minutissima*, and *Scenedesmus obliquus*. The growth of *S*. *capricornutum* and *C*. *minutissima* was shown to be negatively affected by both green macroalgae, while the growth of *S*. *obliquus* was not inhibited. On the other hand, other laboratory studies showed that *C*. *globularis* significantly inhibited the growth of *Scenedesmus* sp. [27]. Studies have confirmed also that marine macroalgae such as *Corallina pilulifera* (Rhodophyta), *Ulva pertusa* (Chlorophyta), *Ishige foliacea* (Ochrophyta), and *Endarachne binghamiae* (Ochrophyta) are capable of secreting allelopathic compounds [19], which inhibit the growth of the toxic and bloom-forming Miozoa *Cochlodinium polykrikoides*. Interestingly, the *C*. *pilulifera* secreted allelopathic compounds that did not inhibit the growth of many other non-toxic microalgae. The inhibitory effect of *Ulva lactuca* on microalgae of the genus *Aureococcus* [28], and the inhibitory effect of the extract of *U. pertusa*, *C. pilulifera*, and *Sargassum thunbergii* on dinoflagellates [29] has been demonstrated. In addition, the effects of *U. petrusa* on *Heterosigma akashiwo* and *Alexandrium tamarense* were investigated [30]. Based on the examples mentioned above, it can be assumed that some macroalgae exhibit diverse allelopathic compounds that depend on both donor and target organism. 

There are also numerous reports on the allelopathic effect of macrophytes on various cyanobacteria species [11,12,14,15,20,31,32,33,34,35,36,37,38,39,40]. Among these studies, allelopathic activity of thallophytes belonging to the genera *Ceratophyllum*, *Myriophyllum*, *Potamogeton*, and *Stratiotes* is the most commonly reported. Mulderij et al. [33] demonstrated the inhibitory effect of the filtrate from *Stratiotes aloides* on two cyanobacteria, the toxic and non-toxic *Microcystis aeruginosa*. Interestingly, a greater negative effect of the filtrate was noted for the toxic strain of tested cyanobacteria. Two years later, Mulderij et al. [34] studied how the extract and filtrate of *S*. *aloides* affected the phytoplankton community. The authors showed that *S. aloides* had both stimulatory and inhibitory effects on phytoplankton assemblages. In addition, the authors noted that inhibition of abundance and biomass was recorded mainly in the case of cyanobacteria. Körner and Nicklisch [11] studied the effect of allelopathic compounds from living tissue of vascular plants *Myriophyllum spicatum*, *Ceratophyllum demersum*, and *Potamogeton pectinatus*. They showed that among cyanobacteria, *Microcystis aeruginosa* was more sensitive than the cyanobacterium *Aphanizomenon flos-aquae*. Moreover, growth inhibition of tested cyanobacteria was positively dependent on *M*. *spicatum* biomass. Experiments on the vascular plant *M. spicatum* were also conducted by Leu et al. [12]. They studied the effect of *M*. *spicatum* extract on the cyanobacteria *Anabaena* sp., *Synechocystis* sp., and *Synechococcus* sp. The allelopathic compounds secreted by this plant inhibited oxygen production in the cyanobacteria *Anabaena* sp. Erhard and Gross [40] studied the effect of extract from two vascular plants: *Elodea canadensis* and *Elodea nuttallii*, on microalgae and cyanobacteria forming natural phytoplankton and phytobenthic communities. Based on the obtained results, Erhard and Gross [40] concluded that allelopathic compounds produced by aquatic plants may reduce the biomass of some organisms, especially cyanobacteria, due to their relatively high sensitivity. On the other hand, Ghobrial et al. [37] showed that the extract of two freshwater vascular plants *Potamogeton pectinatus* and *Ceratophyllum demersum* stimulated the growth of the cyanobacteria *Microcystis aeruginosa* and *Oscillatoria tenuis*. Moreover, Aliotta et al. [39] studied the effect of *Typha latifolia* extract on a wide range of cyanobacteria species (*Nostoc commune*, *Synechococcus leopoliensis*, *Anabaena flos-aquae*, *Phormidium autumnale*, *Porphyrosiphon notarisii*, *Aulosira terrestre*, and *Scytonema hofmanni*). The authors showed that *T*. *latifolia* extract both inhibited and stimulated the growth of tested cyanobacteria. Moreover, the effect was dependent on the target organism.

Compared to the extensive research on allelopathic interactions among aquatic plants mentioned above, knowledge on macroalgal allelopathy on cyanobacteria in aquatic habitats is still fragmentary. Therefore, in this study, two different methods involving the addition of extract and cell-free filtrate obtained from *U*. *intestinalis* were used to determine their effects on the growth, photosynthetic activity, pigment, and toxin content of three of the Baltic bloom-forming cyanobacteria, *Aphanizomenon* sp., *Nodularia spumigena*, and *Nostoc* sp. The study may help to identify the possible role of common macroalgae *U*. *intestinalis* as a significant biological factor influencing harmful cyanobacterial blooms in brackish ecosystems.

## 2. Results

### 2.1. Effect of Ulva intestinalis Extracts and Cell-Free Filtrate on Cyanobacterial Growth

**Figure 1:**
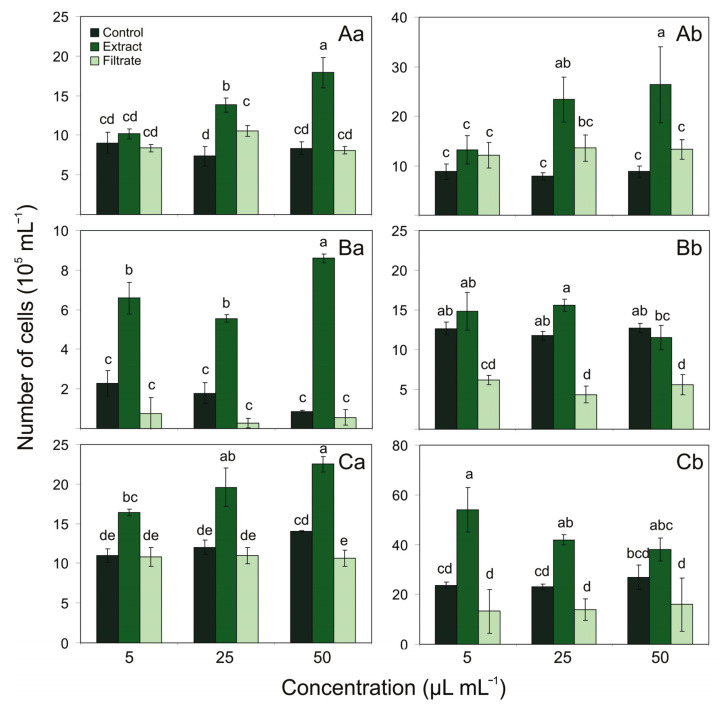
Number of cells (N = 10^5^ cell mL^−1^) for *Aphanizomenon* sp. BA69 (**A**), *N. spumigena* BA15 (**B**), and *Nostoc* sp. BA81 (**C**) for controls and treatments with different concentrations of extract and cell-free filtrate additions obtained from macroalgae *U. intestinalis* after 7 (**a**) and 14 (**b**) days of the expositions (*n* = 3). Different letters indicate significant differences between the means of the treatments (*p* < 0.05, one-way ANOVA). Error bars display the standard deviation.

In order to investigate the effect of *Ulva intestinalis* on the growth of three filamentous cyanobacteria *Aphanizomenon* sp. BA69, *N. spumigena* BA15, and *Nostoc* sp. BA81, the experiments with different concentration of extract and cell-free filtrate additions after 7 and 14 days of the expositions were conducted (Figure 1).

Generally, the addition of aqueous extract of *U. intestinalis* significantly increased the growth of tested cyanobacteria. The growth of *Aphanizomenon* sp. after addition of 25 and 50 µL mL^−1^ of extracts, increased, relative to the control, 187% and 214% after 7 days of the expositions, respectively, and 298% and 299%, respectively, after two weeks of the aqueous extracts’ expositions (Figure 1A). The growth of *N. spumigena* was affected by extract of *U. intestinalis* already at day 7, being, relative to the control, 288%, 312%, and 998% with 5, 25, and 50 µL mL^−1^ additions of the aqueous extracts, respectively (Figure 1B). On the 7th day, the growth of *Nostoc* sp. was, relative to the control, 149%, 162%, and 160% after the 5, 25, and 50 µL mL^−1^ aqueous extracts’ additions, respectively. On the 14th day, the growth of this cyanobacterium was stimulated by 227% and 180% with 5 and 25 µL mL^−1^ additions of the extract, respectively (Figure 1C).

On the other hand, it was shown that the cell-free filtrate addition resulted in a decrease in the number of *N. spumigena* and *Nostoc* sp. cells. In the case of *N. spumigena*, the highest decrease in growth was observed after addition of the 25 µL mL^−1^ of filtrate addition, decreasing by 15% after 7 days of the experiment, relative to the control; however, this result was not statistically significant. However, significant reduction in growth occurred after 14 days. The growth was, relative to the control, 48%, 37%, and 43%, after addition of 5, 25, and 50 µL mL^−1^ of filtrate, respectively (Figure 1B). In experiments with *Nostoc* sp. and *U. intestinalis* cell-free filtrate addition, the negative effect on growth was detected on the 7th day of exposure at 50 µL mL^−1^ and constituted 75% of control (Figure 1C). Surprisingly, as was the case with the extract, the stimulation of the growth of *Aphanizomenon* sp. was noted after addition of the cell-free filtrate obtained from *U. intestinalis*. The growth of *Aphanizomenon* sp. was affected on the 7th day by *U. intestinalis* at 25 µL mL^−1^ of filtrate, being, relative to the control, 143% (Figure 1A).

### 2.2. Effect of Ulva intestinalis Extracts and Cell-Free Filtrate on the Pigments Content

**Figure 2:**
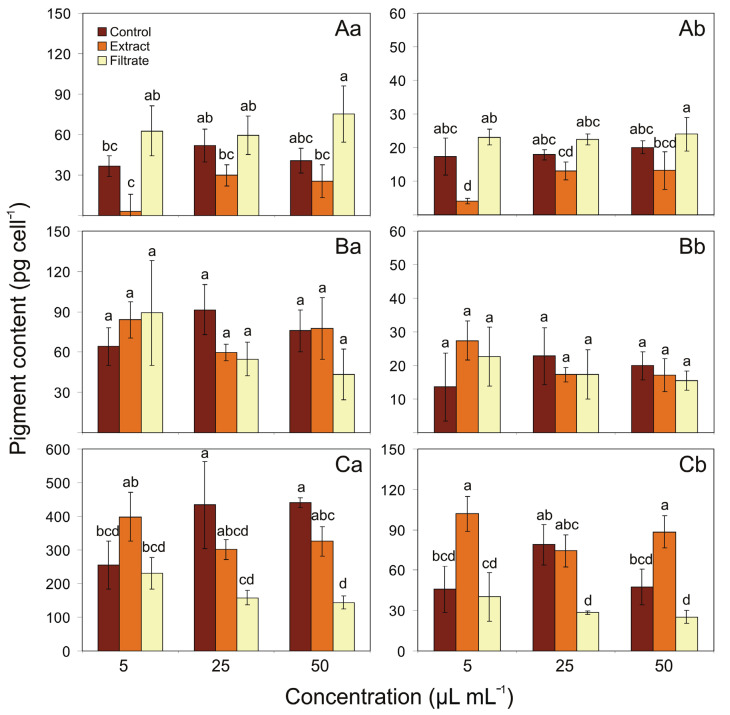
Chl *a* (pg cell^−1^; **a**) and Car (pg cell^−1^; **b**) content for *Aphanizomenon* sp. BA69 (**A**), *N. spumigena* BA15 (**B**), and *Nostoc* sp. BA81 (**C**) for controls and treatments with different concentrations of extract and cell-free filtrate additions obtained from macroalgae *U. intestinalis* after 7 days of the expositions (*n* = 3). Different letters indicate significant differences between the means of the treatments (*p* < 0.05, one-way ANOVA). Error bars display the standard deviation.

The effects of different concentrations of aqueous extract and cell-free filtrate additions obtained from green macroalgae *U. intestinalis* on the Chl *a* and Car content of tested cyanobacteria after 7 days of the expositions are shown in Figure 2. The Car content of *Aphanizomenon* sp. was negatively affected after addition of 5 µL mL^−1^ of extracts, being, relative to the control, 23% (Figure 2A). Surprisingly, addition of extract and filtrate of *U. intestinalis* did not affect Chl *a* and Car content of *N. spumigena* (Figure 2B). In the case of cell-free filtrate addition obtained from *U. intestinalis*, the Chl *a* content in *Nostoc* sp. constituted 36% at 25 µL mL^−1^ and 32% at 50 µL mL^−1^, relative to the control. Furthermore, the significant negative effect on Car content was found for this cyanobacterium in the filtrate addition experiment (at 25 µL mL^−1^), for which it constituted 36% of the control. The Car content of *Nostoc* sp. was also affected after addition of 5 and 50 µL mL^−1^ of extracts, being, relative to the control, 222% and 186%, respectively (Figure 2C).

### 2.3. Effect of Ulva intestinalis Extracts and Cell-Free Filtrate on Fluorescence Parameters

**Figure 3:**
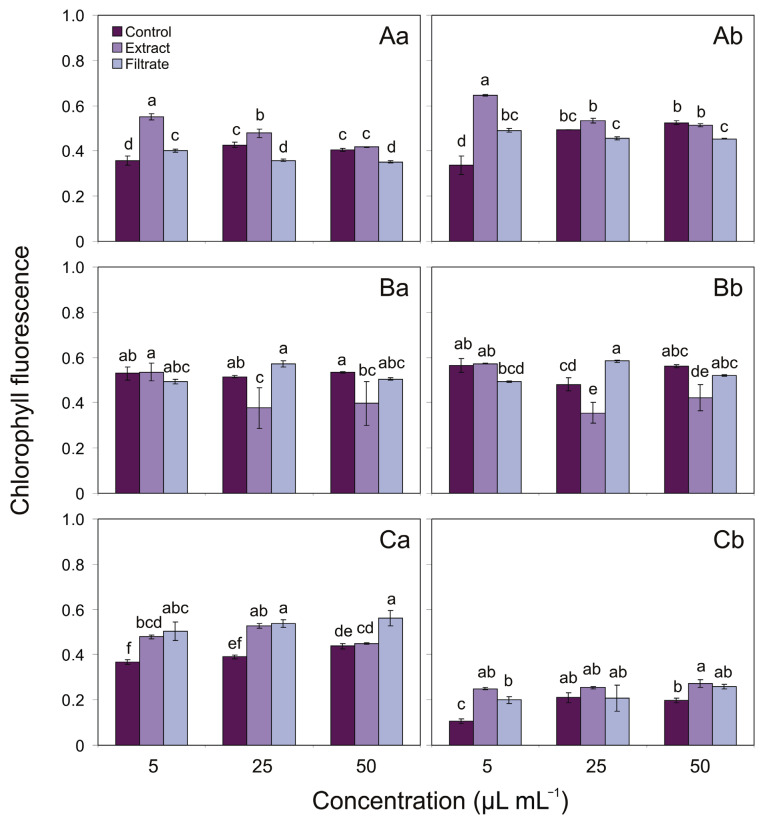
*F*_v_/*F*_m_ (**a**) and ΦPSII (**b**) parameters for *Aphanizomenon* sp. BA69 (**A**), *N. spumigena* BA15 (**B**), and *Nostoc* sp. BA81 (**C**) for controls and treatments with different concentrations of extract and cell-free filtrate additions obtained from macroalgae *U. intestinalis* after 7 days of the expositions (*n* = 3). Different letters indicate significant differences between the means of the treatments (*p* < 0.05, one-way ANOVA). Error bars display the standard deviation.

The effects of different concentrations of aqueous extract and cell-free filtrate additions from *U. intestinalis* after 7 days of the expositions on the maximum quantum yield of PSII photochemistry (*F*_v_/*F*_m_) as well as the effective quantum yield of PSII photochemistry (ΦPSII) in cyanobacterial cells are shown in Figure 3. 

In the extract addition experiments, in *Aphanizomenon* sp., the effect on *F*_v_/*F*_m_ and ΦPSII parameters increased by 154% and 191%, respectively, at the concentration of 5 µL mL^−1^. Moreover, the *F*_v_/*F*_m_ increased by 112%, relative to the level at the control treatment, at the concentration of 25 µL mL^−1^. On the other hand, for cell-free filtrate addition obtained from *U. intestinalis*, the *F*_v_/*F*_m_ in the mentioned cyanobacterium constituted, respectively, 112% at 5 µL mL^−1^, 84% at 25 µL mL^−1^, and 86% at 50 µL mL^−1^, relative to the control. The ΦPSII parameters of *Aphanizomenon* sp., after the addition of 5 and 50 µL mL^−1^ of cell-free filtrate, constituted 145% and 86%, respectively (Figure 3A). Regarding the *F*_v_/*F*_m_ parameter, for *N. spumigena* in the extract addition assay, it was 73% at a concentration of 25 µL mL^−1^ and 74% at a concentration of 50 µL mL^−1^, relative to the control. Furthermore, in *N. spumigena*, the significant decrease in the ΦPSII parameter was observed after 25 and 50 µL mL^−1^ of extract addition, being 73% and 75%, respectively. For this same species, it was found that after addition of 25 µL mL^−1^ of macroalgal filtrate concentrations, the values of ΦPSII constituted 121% (Figure 3B). Interestingly, the addition of extract and filtrate obtained from *U. intestinalis* significantly increased the fluorescence parameters of *Nostoc* sp. It was found that the additions of 5 and 25 µL mL^−1^ of extract concentrations resulted in an increased *F*_v_/*F*_m_ for this cyanobacterium after the seventh day of exposure, i.e., increases of 130% and 135%, respectively, compared with control. Moreover, the addition of macroalgal filtrate at the concentration of 5, 25, and 50 µL mL^−1^ resulted in an increased *F*_v_/*F*_m_ value for *Nostoc* sp. after 1 week of exposure (by 137%, 137%, and by 127%, respectively). The ΦPSII of *Nostoc* sp. was positively affected after addition of 5 and 50 µL mL^−1^ of extracts, being, relative to the control, 239% and 137%, respectively. In the cell-free filtrate addition experiments, the values of ΦPSII for the mentioned cyanobacterium were significantly different from the control at the concentration of 5 µL mL^−1^, when it constituted 202% (Figure 3C).

### 2.4. Effect of Ulva intestinalis Extracts and Cell-Free Filtrate on Microcystin and Phenol Content

The effects of 50 µL mL^−1^ of extract and cell-free filtrate concentrations obtained from *U. intestinalis* after 7 days of the expositions on the MC-LR and phenol content in target cyanobacteria cells are shown in Table 1. Furthermore, toxin and phenol content in cyanobacterial culture were reported in the Supplementary Material (Figure S1, Table S1).

It was shown that the addition of aqueous extract obtained from *U. intestinalis* caused a significant decrease in the MC-LR content in *Nostoc* sp. cells and was about 18% compared to the control. On the other hand, the addition of the filtrate caused a slight decrease in the content of this toxin, but it was not statistically significant. Moreover, it was noted that two other cyanobacteria, *Aphanizomenon* sp. BA69 and *N. spumigena* BA15, were unable to produce MC-LR (Table 1).

It was also demonstrated that all tested cyanobacteria were capable of producing phenols (Table 1). Moreover, it was shown that the addition of the extract and filtrate obtained from *U. intestinalis* after 7 days of the expositions had a significant effect on the cell-specific phenol content of the examined cyanobacteria. It was found that the addition of the extract increased the phenol content in *Aphanizomenon* sp., *N. spumigena*, and *Nostoc* sp. cells, which was about 4.0-fold, 1.6-fold, and 5.8-fold higher, respectively, than the control treatments. The addition of the cell-free filtrate from *U. intestinalis* also increased the cell-specific phenol content of *N. spumigena*, which was about 160%. Surprisingly, the addition of the cell-free filtrate caused a significant decrease (38%) in the phenol content in *Aphanizomenon* sp. cells.

## 3. Discussion

### 3.1. Allelopathic Activity of the Extract Obtained from U. intestinalis

The present study investigated the allelopathic effects of aqueous extract and cell-free filtrate from the Baltic green macroalgae *U*. *intestinalis* on the growth and the physiology of three bloom-forming cyanobacteria: *Aphanizomenon* sp., *N. spumigena*, and *Nostoc* sp. In this study, the number of cyanobacteria was measured after 7 and 14 days in order to see how these organisms respond to allelopathic compounds produced and released by the macroalga after a longer exposure time. The results showed that extracts obtained from freshly harvested material had a stimulating effect on the tested cyanobacteria strains after the 7 days of the exposition. However, after the two weeks of the experiment, the stimulation was no longer as high or had no significant effect on the cyanobacterial growth. Extending the experiment time may have a negative effect on cell physiology due to the aging of the tested organism. Therefore, the allelopathic effect of the analyzed green macroalga on the photosynthesis performance as well as the pigments and toxin content were analyzed only after one week of exposure. In the case of the experiment on *N*. *spumigena*, there was even a 15-fold increase in the number of cells in the sample after one week. In the case of the *Nostoc* sp. growth, a significant stimulating effect of the extract additions after 7 and 14 days of the experiment was also demonstrated. Moreover, it was found that the green macroalgae extract at the lowest concentration (5 µL mL^−1^) had no significant effect on the growth of *Aphanizomenon* sp. However, at higher concentrations (25 and 50 µL mL^−1^), stimulation was observed up to 187% and 214%, respectively, relative to the control. In this study, we also showed that allelopathic compounds produced by Baltic macroalgae *U*. *intestinalis* can affect the pigment content and photosynthetic activity of target cyanobacteria. Generally, it was shown that the extract of *U. intestinalis* had no effect on the Chl *a* content of tested cyanobacteria. It was also shown that the Car content of *Aphanizomenon* sp. and *Nostoc* sp. was significantly changed under the influence of the extract obtained from green macroalga *U*. *intestinalis*. On the other hand, the addition of the extract had no effect on the Car content of *N*. *spumigena*. The Car content of *Aphanizomenon* sp. was negatively affected after addition of the lowest extract concentration. Surprisingly, the Car content of *Nostoc* sp. was also positively affected after addition of 5 and 50 µL mL^−1^ of extracts obtained from *U*. *intestinalis*. Moreover, in our work we showed that fluorescence parameters of *Nostoc* sp. were stimulated by adding the extract obtained from donor macroalgae. For *N. spumigena*, the significant decrease in *F*_v_/*F*_m_ and ΦPSII parameters were observed after 25 and 50 µL mL^−1^ of extract addition. Furthermore, it was shown that higher concentrations of extract inhibited the *F*_v_/*F*_m_ and ΦPSII of *Aphanizomenon* sp., while low concentrations stimulated these parameters. 

Wang et al. [29] noted that high concentrations of aqueous extracts of *U*. *pertusa* strongly inhibited microalgae growth and caused cell degradation of *H*. *akashiwo* and *A*. *tamarense*. At lower extract concentrations, *U*. *pertusa* did not cause mortality of the test organisms and even stimulated their growth. This was probably due to the fact that at higher concentrations, all the microalgae cells were degraded by the allelopathic substances in a short time interval, whereas at lower concentrations, some microalgae cells were still alive after the degradation of some of the allelopathic compounds, allowing the remaining target cells to grow by providing them with nutrients from the dead microalgae cells. Similar observations were also made by Jin and Dong [30] when studying the effect of *U*. *pertusa* on the growth of *H*. *akashiwo*. Jin and Dong [30] showed that *U*. *pertusa* extract at relatively higher concentrations was lethal to *H*. *akashiwo*, while the extract at lower concentrations either had no effect on algal growth or significantly stimulated it. In this study, the stimulation of the growth of Baltic cyanobacteria *Aphanizomenon* sp., *N. spumigena*, and *Nostoc* sp. was also noted due to the addition of *U. intestinalis* extract. Thus, it is possible that the stimulation of cyanobacteria in the present study was due to an increase in nutrients in the medium that were contained in the *U*. *intestinalis* thallus and were extracted upon addition of the aqueous extract. In this study, cyanobacterial species were cultivated in the f/2 medium. However, some authors indicated that the f/2 medium is not suitable for cyanobacterial culture [41]. On the other hand, there are studies that confirm that cyanobacteria achieved the highest biomass on the f/2 medium [42]. Regardless of the medium used, it is possible that nutrient enrichment from the macroalgal extract may provide essential growth-related nutrients to cyanobacterial cells. Thus, the allelopathic effects of macroalgae on cyanobacteria maintained in various culture media should be investigated in future research.

In turn, Jin et al. [43] conducted laboratory experiments under controlled environmental conditions to investigate the allelopathic effects of *U*. *pertusa* and *Ulva linza* on *Prorocentrum micans* cultures. Aqueous and methanolic extracts from both *Ulva* species showed strong growth inhibitory effects on *P*. *micans*, while extracts from less polar organic solvents had no visible effect. This suggests that the extracted allelopathic compounds had relatively high polarity. It is noteworthy that methanolic extracts from both *Ulva* species showed the highest allelopathic activity. Based on the results, the authors concluded that the methanolic extracts were about 50 times more effective than the aqueous extracts. In the present study, we used aqueous extracts from *U*. *intestinalis*, so it can be assumed that the use of other extracts from this Baltic green macroalga could significantly enhance the observed effect.

### 3.2. Allelopathic Activity of the Filtrate Obtained from U. intestinalis

In this work, a stimulatory allelopathic effect of the filtrate obtained from *U*. *intestinalis* on the cyanobacterium *Aphanizomenon* sp. and an inhibitory effect for *N*. *spumigena* and *Nostoc* sp. were demonstrated. The number of *Aphanizomenon* sp. cells increased under the *U*. *intestinalis* filtrate additions in the first week of the experiment. It was shown that after the two weeks of the experiment, for each of the three concentrations, a decrease in the number of cells of *N. spumigena* was observed. In the case of *Nostoc* sp. growth, the inhibitory effect of the green macroalgae filtrate was demonstrated only after 7 days of the experiment. However, it should be noted here that not every concentration of the filtrate contributed to a change in the number of cyanobacteria cells in the experiment. Moreover, in this study, reduction in cell-specific Chl *a* content was noted only for *Nostoc* sp. upon addition of the macroalgal cell-free filtrate. The addition of the filtrate had no effect on the Chl *a* and Car content of *Aphanizomenon* sp. and *N*. *spumigena*. On the other hand, the significant negative effect on Chl *a* and Car content was found for *Nostoc* sp. in the filtrate addition experiment. In addition, the effects of filtrate from the green alga *U*. *intestinalis* on the values of fluorescence parameter *F*_v_/*F*_m_ and ΦPSII for *Aphanizomenon* sp., *N*. *spumigena*, and *Nostoc* sp. were demonstrated. It was found that fluorescence parameters of *N*. *spumigena* and *Nostoc* sp. were stimulated by adding the cell-free filtrate. On the other hand, the low concentrations stimulated fluorescence parameters of *Aphanizomenon* sp., while higher concentrations significantly inhibited the *F*_v_/*F*_m_ and ΦPSII.

Nan et al. [44] showed that the filtrate obtained from *U*. *pertusa* culture showed no inhibitory effect on the growth of seven microalgae species (*Heterosigma akashiwo*, *Skeletonema costatum*, *Tetraselmis subcordiformis*, *Nitzschia closterium*, *Chaetoceros gracile*, *Isochrysis galbana*, and *Alexandrium tamarense*). However, this filtrate addition strongly inhibited the growth of *Chroomonas placoidea*. Jin and Dong [30] also showed that single and multiple additions of *U*. *pertusa* filtrate had no inhibitory effect on the growth of two microalgae *H*. *akashiwo* and *A*. *tamarense*. On the other hand, Nakai et al. [31] showed that growth inhibition of the cyanobacterium *M*. *aeruginosa* by the macrophyte *Myriophyllum spicatum* required the continuous secretion of certain unstable growth-inhibiting allelopathic compounds. They also found that *M*. *aeruginosa* growth was not significantly inhibited after a single addition of allelopathic compounds, whereas multiple additions of metabolites from *M*. *spicatum* had an inhibitory effect on cyanobacterial growth. The same phenomena were also observed between the macroalgae *Chara aspera* and the green alga *Scenedesmus acutus* [45], where the reduction of *S*. *acutus* growth occurred only when *C*. *aspera* was actually present in the co-culture experiment. Jin and Dong [30] and Wang et al. [29] suggested that macroalgae from the genus *Ulva* releases rapidly decomposing allelopathic substances, and continuous allelochemical release from fresh thallus is necessary for effective inhibition of microalgal growth. Thus, the reported lack of effect of some concentrations of *U. intestinalis* filtrate on the cyanobacterial growth in our work may indicate that the amount of rapidly degradable allelopathic compounds released into the medium by this donor macroalgae was insufficient to detect a visible and significant response. Moreover, the increased potency of the extracts may also suggest that the allelopathic compounds in the cells were more concentrated than the portion released via cell-free filtrate into the environment.

The most common effect of allelopathic compounds produced by macroalgae and aquatic macrophytes recorded in the environment is the inhibition of growth of associated cyanobacterial and microalgal species (e.g., [20,28]). However, there are many mechanisms that may be responsible for stimulating or inhibiting the growth of target cyanobacteria by producing active metabolites by macroalgae into the environment. Inhibition of photosystem II (PSII) and changes in pigment content of target organisms by compounds secreted by macroalgae and aquatic macrophytes have been rarely studied in the literature. Körner and Nicklisch [11] studied the effects of the aquatic macrophytes *M*. *spicatum*, *Ceratophyllum demersum*, and *Potamogeton pectinatus* on photosynthesis of the cyanobacteria *M*. *aeruginosa* and *Aphanizomenon* sp. They showed inhibition of PS II in *M*. *aeruginosa*. PSII of the cyanobacterium *Aphanizomenon* sp. did not show changes under the influence of allelopathic compounds secreted by the tested macrophytes. Złoch et al. [46] studied the allelopathic activity of *C*. *aspera*, *C*. *baltica* and *C*. *canescens* on the fluorescence of the cyanobacterium *Synechococcus* sp. They showed that the photosynthetic parameters of the cyanobacterium were significantly changed. They found that after the first week of the experiment the photosynthetic values of *Synechococcus* sp. after the addition of the extract obtained from *C*. *aspera* were significantly lower than the control culture. A decrease in fluorescence was also noted in the case of *C*. *baltica* extracts. Only an extract concentration equal to 5 μL mL^−1^ of *C*. *baltica* and *C*. *canescens* caused an increase in fluorescence for *Synechococcus* sp. after the seventh day of exposure. A detailed study on phytoplankton was also conducted by Prince et al. [47], who analyzed the effect of *K*. *brevis* on photosynthetic performance based on the fluorescence parameter *F*_v_/*F*_m_. The study showed that the target organisms (*Akashiwo* cf. *sanguinea*, *Amphora* sp., *Asterionellopsis glacialis*, *P*. *minimum*, and *S*. *costatum*) showed a decrease in photosynthetic efficiency after 1 h of exposure to the extract obtained from *K*. *brevis*. A strong decrease in PSII productivity as influenced by the addition of the extract was recorded in *S*. *costatum*, which was 68% compared to the control. Photosynthetic efficiency in *Akashiwo* cf. *sanguinea*, *Amphora* sp., *A*. *glacialis*, and *P*. *minimum* was inhibited between 19% 43% relative to the control. Moreover, the inhibition of photosynthetic efficiency by the extract obtained from *K*. *brevis* coincided with the negative effect on the growth of the target organisms tested. It is believed that measuring PSII efficiency can provide information on when target organisms are most susceptible to allelopathic effects. However, it is worth noting here that organic carbon sources including monosaccharide, disaccharide, oligosaccharide, and polysaccharides can be absorbed by microalgal [48,49,50] and cyanobacterial [51] cells, and this heterotrophic growth condition mostly influences chlorophyll contents [48,52,53]. Therefore, changed photosynthetic pigment contents and the maximum quantum yield of PSII photochemistry (*F*_v_/*F*_m_) as well as the effective quantum yield of PSII photochemistry in cyanobacteria cells (ΦPSII) can be changed by the presence of available organic carbon sources [53]. Based on the obtained results, the present study demonstrated that the Baltic macroalgae affected photosynthetic pigment content and, thus, photosynthesis performance in target cyanobacteria, indicating a potential mechanism of action of the allelopathic compounds they produced. Reduction in photosynthetic pigment content and inhibition of PSII may result in lower primary production, resulting in slower growth rates of associated cyanobacterial species.

An interesting issue is the stimulation of the growth of *Aphanizomenon* sp. after addition of cell-free filtrate obtained from *U*. *intestinalis*. Stimulation of *Aphanizomenon* sp. growth under the influence of allelopathic compounds secreted by donor cyanobacteria was also reported by Śliwińska-Wilczewska et al. [54] and Konarzewska et al. [55]. *Aphanizomenon* sp. is known to create annual harmful blooms in the Baltic Sea [56]. It is possible that this cyanobacterium is capable of benefiting from allelochemicals produced by certain donor organisms. Such a skill would give *Aphanizomenon* sp. a competitive advantage over other Baltic cyanobacteria. This is a very interesting issue that requires further detailed research. On the other hand, the present study also showed that the addition of filtrate from *U*. *intestinalis* significantly inhibited the growth of *N*. *spumigena* and *Nostoc* sp. This indicates that the concentration of allelopathic compounds produced by Baltic *U*. *intestinalis* may be higher than that of marine and freshwater *U*. *pertusa* and *U*. *lactuca* investigated by Nan et al. [44], Jin and Dong [30], and Wang et al. [29]. The above-mentioned studies showed the possibility of secretion of unstable allelopathic compounds inhibiting the growth of selected organisms not only in freshwater and marine environments but also in brackish ecosystems.

### 3.3. Effects of Ulva intestinalis on Cyanobacterial MC-LR and Phenol Content 

In this work, the effects of aqueous extract and cell-free filtrate concentrations obtained from *U. intestinalis* on the MC-LR and phenol content in target cyanobacteria cells were also demonstrated. It was shown that the addition of extract obtained from *U. intestinalis* caused a significant decrease in the MC-LR content in *Nostoc* sp. cells. Moreover, the addition of the filtrate also caused a slight decrease in the content of this toxin; however, it was not statistically significant. In our work we also demonstrated that that the addition of the extract from *U. intestinalis* had a positive effect on the cell-specific phenol content of all the examined cyanobacteria. Moreover, it was found that the addition of the cell-free filtrate obtained from *U. intestinalis* increased the phenol content in *N. spumigena* cells. The only exception was *Aphanizomenon* sp., where the cell-specific phenol content decreased due to the addition of the filtrate. Various factors can affect the MC [57] and phenol content of cyanobacterial cells [58]. In addition, MC is a known toxin affecting the ecosystem, from microalgae, zooplankton, fish, birds, and mammals [59]. In addition, people consume organisms that are capable of bioaccumulating MC, such as mussels, crayfish, crabs, or simply by drinking water directly contaminated with this toxin [59]. Phenols have been shown to negatively affect microbial activity for a number of different microorganisms [60,61,62,63,64,65], exemplifying the toxicity of such compounds. Furthermore, there are several toxic phenolic compounds that are on the priority pollutant list [66]. In cyanobacteria, MC and phenol content can be changed by manipulating different culture conditions [58,67]. In our work, we showed that *U*. *intestinalis* can modulate the content of harmful metabolites in cyanobacterial cells due to the production of allelopathic compounds. It is commonly known that selected cyanobacteria produce numerous secondary metabolites (e.g., [9,68,69,70]). Since demonstrating a number of chemical compounds on cyanobacteria produced by cyanobacteria requires thorough preparation and time-consuming experiments, we decided to select only two, but very important compounds. MC and phenols were selected as ‘representatives’ of the compounds, with a negative effect impact on coexisting plants and animals [2]. Moreover, MC-LR is listed as one of the most important algal toxins [71], that is especially harmful both for humans and animals. What is more, the bioaccumulation of MC in water organisms is documented [72], while phenol and its derivatives are one of the largest groups of environmental pollutants, that exhibit a wide range of biological effects, e.g., affecting both the activity of enzymes and photosynthetic processes of marine organisms [73]. Importantly, phenol due to its microbial toxicity accumulates in contaminated soils [74]. At this point we want to add that our research, focusing exclusively on MC and phenols, is particularly important both for the aquaculture production and environmental pollution. This research on selected cyanobacteria after exposure to macroalgal allelochemicals is pioneering and therefore definitely needs to be continued. We believe that the obtained results will be interesting for researchers and will contribute to the further development of research in this field. 

### 3.4. Ecological Significance of Allelopathic Effects of Baltic Macroalgae Ulva sp.

Although macroalgal blooms are widespread and negatively impact many aquatic ecosystems, they have been largely neglected in biodiversity conservation research. The effects of macroalgal green algal blooms (commonly referred to as “green tide”) are diverse and have been summarized by Fletcher [75] and Ansell et al. [76]. It is worth mentioning here that *U*. *prolifera*, the species causing the largest green tide in the world, is widely distributed in intertidal zones and estuaries worldwide due to its tolerance to a wide range of salinities and water temperatures, high growth rate and remarkable growth ability [77]. This species was also found in the Baltic Sea [78]. In a study conducted by Xu et al. [17] found that allelopathic compounds produced by *U*. *prolifera* can be actively released into the environment. In the study by Xu et al. [17] the highest concentration of *U*. *prolifera* was 3.75 g wet weight per liter of the culture. However, the authors noted that the effect of higher concentrations of *U*. *prolifera* should be investigated in the future, as higher concentrations may have a significant impact on the observed effects. In the present study, three concentrations were used to investigate the allelopathic effect for each of two experimental methods used. For the aqueous extract and filtrate-based experiments, 5, 25, and 50 µL mL^−^^1^ were added to the target culture. Based on the results recorded in this study, it was found that the allelopathic effect of the extract stimulated the growth of tested cyanobacteria. The filtrate inhibited the growth of cyanobacteria *N*. *spumigena* and *Nostoc* sp. In contrast, the number of *Aphanizomenon* sp. cells was stimulated by the filtrate obtained from *U*. *intestinalis*. Generally, it was observed that the higher the concentration, the stronger the effect was obtained, confirming the observations of Xu et al. [17]. The present study may represent a significant advance in the study of ecological questions concerning the effects of green tides of *Ulva* sp. on cyanobacterial communities. It has been also demonstrated that *U*. *intestinalis* can produce a wide range of metabolites that have negative effects on other organisms. It has been shown that *Ulva* sp. may produce: penostatins A-H [79], cytochalasans, penochalasins A-H [80], chaetoglobosin [81], and communesins A-B [82]. Furthermore, based on previous studies conducted on Asian species belonging to *Ulva*, some of the allelopathic compounds produced by these green macroalgae may contain polyunsaturated fatty acids [83,84]. The work of Tang and Glober [28] also showed that the effect of concentrated extract of *U*. *lactuca* was as strong or even stronger than that of the filtrate, which is consistent with the hypothesis that polyunsaturated fatty acids may be active allelopathic agents. Hence, our next work will focus on the characterization of allelopathic compounds produced by the Baltic *U.* *intestinalis* responsible for stimulating and inhibiting the growth of bloom-forming cyanobacteria.

On the other hand, macroalgae belonging to the genus *Ulva* are widely distributed; thus, harvesting these macroalgae from shorelines provides an easy, economical and environmentally friendly way to potentially control the species responsible for creating massive blooms [19]. Jin et al. [43] in their work showed that two green algae, *U*. *pertusa* and *U*. *linza*, can secrete allelopathic substances that can inhibit the growth of *Prorocentrum micans*, which are known to create harmful blooms in the marine environment. Furthermore, Tang and Glober [28] showed that *U*. *lactuca*, through the secretion of allelopathic compounds, is able to limit the growth of seven phytoplankton species (*Aureococcus anophagefferens*, *Chattonella marina*, *Cochlodinium polykrikoides*, *Karlodinium veneficum*, *K*. *brevis*, *P*. *minimum*, and *Pseudo*-*nitzschia multiseries*) that are known to form massive blooms worldwide. However, it is worth emphasizing here that the application of allelopathic compounds for the control of cyanobacterial blooms may require their identification, which is largely difficult. Further research is also needed to clarify the mechanism of selective modes of action of allelopathic compounds against specific target organisms. Although it is possible to control algal growth under laboratory conditions, in natural ecosystems the effects of allelochemicals could be very different [31]. Therefore, more research should be conducted on the allelopathic activity of bioactive macroalgal metabolites on a wide range of target organisms in order to control cyanobacterial blooms in coastal areas in the future.

## 4. Conclusions

Although harmful properties are rarely associated with macroalgae, there is increasing evidence that some green macroalgae and their secondary metabolites have allelopathic properties [85]. In this study, we demonstrated that *U*. *intestinalis* effectively inhibits the growth of Baltic cyanobacteria *Nodularia spumigena* and *Nostoc* sp. Thus, the results obtained in this study provide new insight into the ecological role of macroalgae *U*. *intestinalis* in reducing the occurrence of certain massive bloom-forming cyanobacteria species in the Baltic Sea region. On the other hand, we also found positive allelopathic effects of *U. intestinalis* on other bloom-forming and toxic cyanobacterium *Aphanizomenon* sp. These results may indicate that in areas where there are underwater meadows and/or large biomass of *U. intestinalis*, may appear *Aphanizomenon* sp. in large quantities. Green macroalgae *U*. *intestinalis* are widely distributed and found in many aquatic ecosystems. Our results provide insight into the interactions between macroalgae and cyanobacteria in coastal areas and indicate the need to isolate and characterize allelopathic substances produced by Baltic green algae of the genus *Ulva* in the future. Chemical interactions between macroalgae and other organisms may play an important role in their dominance in coastal areas. Collection and processing of macroalgae biomass to predict and control Cyanobacterial Harmful Algal Blooms (CyanoHABs) [19] events would contribute to the development of the local economy.

## 5. Materials and Methods

### 5.1. Material and Culture Conditions

**Figure 4:**
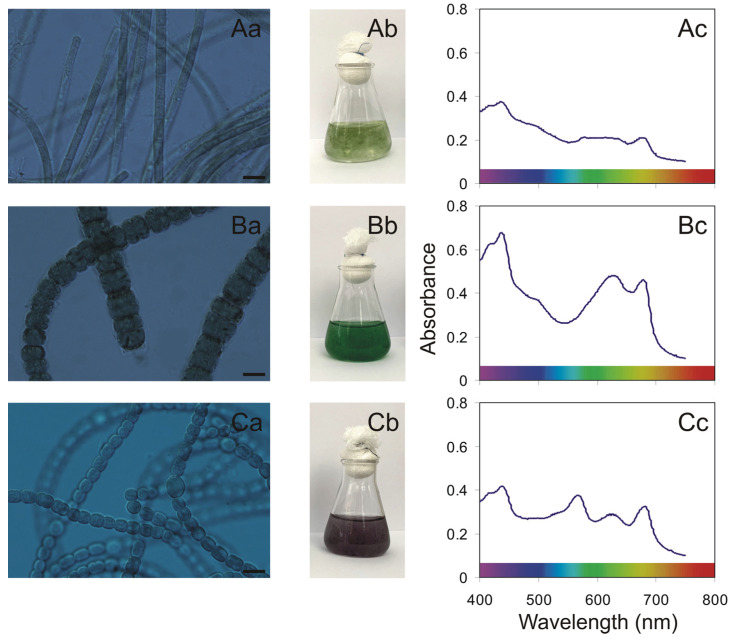
Light microscope photographs of *Aphanizomenon* sp. BA69 (**A**), *N. spumigena* BA15 (**B**), and *Nostoc* sp. BA81 (**C**). ((**a**); scale = 10 μm). photographs of the cyanobacterial culture in 25-mL glass flasks from the ex-perimental phase (**b**); and PAR absorption spectra determined for this strain at an optical density (OD750) = 0.1 (**c**).

The experiment was conducted with the cyanobacteria *Aphanizomenon* sp. BA69, *Nodularia spumigena* BA15, and *Nostoc* sp. BA81 isolated from the southern Baltic Sea region (Figure 4). These strains were maintained as the non-axenic batch culture in the Culture Collection of Baltic Algae (CCBA) at the University of Gdańsk, Poland (http://ccba.ug.edu.pl/pages/en/home.php, accessed on 20 August 2021). 

Cyanobacterial strains (V = 20 mL) were maintained in 25-mL glass Erlenmeyer flasks at 18 °C, 10 μmol photons m^−2^s^−1^ (16:8 h light:dark cycle), and salinity 7.5 psu. Experiments were conducted in incubators capable of maintaining constant temperature condition (±1 °C). Fluorescent lamps (Cool White 40W, Sylvania, OH, USA) were used as a source of irradiance. The culture medium employed was f/2 [86] which have been prepared from water taken from the Baltic Sea with salinity of 7.5 psu and autoclaved. These growth conditions were used for both control and allelopathic experiments. The photosynthetically active radiation (PAR) was measured using a spherical quantum-meter (LI-COR, Lincoln, NE, USA). The salinity was verified by salinometer (inoLab Cond Level 1, Weilheim in Oberbayern, Germany).

*U. intestinalis* was selected based on the allelopathic potential of species from the genus *Ulva* [28,29,30,41]. This green macroalgae was collected manually from the coastal region of the southern Baltic Sea, Poland (54°31′08″ N and 18°31′54″ E), immediately transported to the laboratory, and carefully washed with distilled water to remove attached organisms according to a method proposed by Mabrouk et al. [87]. In the next step, *U. intestinalis* was placed in a glass aquarium (V = 6 L) with the sterilized f/2 medium (V = 5 L) for two days. After that time, this *U. intestinalis* was taken to carry out allelopathic experiments (both for obtaining the extract and the cell-free filtrate). Determination of *U. intestinalis* was based on the examination of morphological features. Herbarium sheets were prepared in accordance with guidelines in Rybak [88]. Currently, the herbarium sheets were deposited at the Institute of Oceanography, University of Gdańsk, Poland and are available for inspection on the website (https://zielnik.ug.edu.pl/en/home/ accessed on 20 August 2021).

### 5.2. Experimental Setup

The allelopathic effects of *U. intestinalis* were tested according to a method proposed by Wang et al. [29] for cell-free filtrate additions and Złoch et al. [46] for aqueous extract addition. The cyanobacteria monocultures were exposed to three different concentrations of aqueous extracts and cell-free filtrate of *U. intestinalis*. All allelopathic tests were conducted in triplicate.

To prepare the aqueous extract, the air-dried materials were homogenized in a mortar grinding machine. For the bioassay experiment, 50 g of dried *U. intestinalis* was extracted with 20 mL of autoclaved f/2 medium for 10 min. The aqueous extract of *U. intestinalis* was filtered through glass fiber filters (Whatman GF/C, Saint Louis, MO, USA) using a vacuum pump (0.5 bar). Sterilized Erlenmeyer flasks (V = 25 mL) contained 20 mL of f/2 medium with target cyanobacteria strains (initial inoculum was 10^5^ cells mL^−1^) and different volumes of macroalgae extract treatments. Experimental treatments were prepared by adding 100, 500 and 1000 µL of *U. intestinalis* extracts to 25 mL Erlenmeyer flasks containing 20 mL of cell suspensions of the targeted cyanobacteria (the final concentrations of extract were: 5, 25, and 50 µL mL^−1^). Controls consisted of the addition of 100, 500 and 1000 µL of filtrated f/2 medium to 25 mL Erlenmeyer flasks containing 20 mL of the same cyanobacteria strains. The flasks with cyanobacteria were gently swirled once a day.

To prepare the cell-free filtrate, a different concentration of f/2 medium in which *U. intestinalis* was grown for 3 days in Erlenmeyer flasks (V = 250 mL) at a concentration of 80 g wet weight L^−1^ was added. This filtrate was enriched with nutrients equivalent to the f/2 medium and filtered through a membrane filter (Whatman GF/C, Saint Louis, MO, USA). Treatments were prepared by adding 100, 500 and 1000 µL of *U. intestinalis* exudates to 20 mL of target cyanobacteria. Initial inoculum of cyanobacteria strains was 10^5^ cells mL^−1^ and the final concentrations of cell-free filtrate were: 5, 25, and 50 µL mL^−1^. Controls consisted of the addition of 100, 500 and 1000 µL of filtrated f/2 medium to 20 mL of the same target cyanobacteria strains. The flasks with cyanobacteria were gently swirled once a day.

### 5.3. Determination of the Culture Density

Cell densities in the cyanobacteria cultures were estimated with previously determined linear regression models between the optical density (OD) and the number of cells (N mL^−1^) following a procedure according to Śliwińska-Wilczewska et al. [54] for *Aphanizomenon* sp. and *Nostoc* sp. and Barreiro Felpeto et al. [89] for *N. spumigena*. N was counted using a Bürker chamber [90]. OD was measured spectrophotometrically at 750 nm with a Multiskan GO UV-VIS spectrophotometer (Thermo Scientific, Waltham, MA, USA). Cyanobacterial cell densities were examined for control and experiments with aqueous extract and cell-free filtrate from *U. intestinalis* additions after 7 and 14 days of the experiment.

### 5.4. Determination of the Pigments Content

The Chl *a* and Car content of analyzed cyanobacteria were measured by the spectrophotometric method [54]. After 7 days of incubation, 15 mL of culture was filtered using membrane filters (Whatman GF/C, Saint Louis, MO, USA) to separate the cyanobacteria cells from the medium. Chl *a* and Car were extracted from the cyanobacteria cells with 90% acetone (V = 2 mL). The pigment extract was held in the dark for 2 h at −20 °C and centrifuged at 12,000 rpm for 2 min to remove filter particles (Sigma 2-16P, Osterode am Harz, Germany). The extinction was determined with a Thermo Scientific UV-VIS Multiscan GO spectrophotometer (Thermo Scientific, Waltham, MA, USA) using a 1 cm glass cuvette. The concentrations of Chl *a* and Car were calculated according to Strickland and Parsons [91] with the formula: Chl *a* (μg mL^−1^) = 11.236(A_665_ − A_750_)V_a_/V_b_, and Car (μg mL^−1^) = 4(A_480_ − A_750_)V_a_/V_b_, where: V_a_—extract volume (mL) and V_b_—sample volume (mL). A_n_—absorption at a specific wavelength; _n_—wavelength (nm).

### 5.5. Measurements of the Photosynthesis Performance

Chlorophyll *a* fluorescence was measured using PAM method by a fluorometer (FMS1, Hansatech, King’s Lynn, UK) according to the method described by Złoch et al. [46]. Tested cyanobacteria were taken for chlorophyll fluorescence analysis after the 7th day of the experiment. The maximum quantum yield of PSII photochemistry in cyanobacterial cells was obtained as *F*_v_/*F*_m_ by measuring the maximum fluorescence (*F*_m_) and minimum fluorescence (*F*_0_): *F*_v_/*F*_m_ = (*F*_m_ − *F*_0_)*F*_m_^−1^, where *F*_m_ and *F*_0_ are the maximum and minimum fluorescence in dark-adapted cells, respectively. The effective quantum yield of PSII photochemistry in cyanobacteria cells was obtained as ΦPSII by measuring the maximal fluorescence in the light-adapted state (*F*_m’_) and steady-state fluorescence (*F*_s_): ΦPSII = (*F*_m’_ − *F*_s_) *F*_m’_^−1^ [92].

### 5.6. Measurements of the Microcystin and Phenol Content

ELISA quantitation for microcystin-LR (MC-LR) equivalents was performed using a Microcystin-LR ELISA kit (colorimetric) (Abnova, Taipei, Taiwan) as per the manufacturer’s specifications [93]. Final absorbances were read at 450 nm in triplicates using a Thermo Scientific Multiscan Go microplate reader (Thermo Scientific, Waltham, MA, USA). Target cyanobacteria were taken for MC-LR analysis after the 7th day of the experiment for controls and allelopathic treatment with the additions of 50 µL mL^−1^ of extracts and cell-free filtrate from *U. intestinalis*.

Phenols were quantified in the liquid phase by using a cuvette test (0.05–5.0 mg/L, 24 tests) from Hach Lange (LCK 346 Phenols, Hach Lange, Düsseldorf, Germany) according to the manufacturer’s specifications [94]. In this method, phenols react with 4-nitroaniline to form a yellow color complex, which was measured in a Thermo Scientific Multiscan Go microplate reader (Thermo Scientific, Waltham, MA, USA). The effects of 50 µL mL^−1^ of extract and cell-free filtrate concentrations obtained from *U. intestinalis* after the 7th day of the expositions on the phenol content in target cyanobacteria cells were determined.

### 5.7. Statistical Analyses

One-way ANONA was applied to determine whether the growth of the analyzed cyanobacteria *Aphanizomenon* sp. BA69, *Nodularia spumigena* BA15, and *Nostoc* sp. BA81 (after 7 and 14 day of the experiment), pigment content (Chl *a* and Car) and fluorescence parameters (*F*_v_/*F*_m_ and ΦPSII) when treated with different concentration of the extracts and cell-free filtrate of *U. *intestinalis** differed from the control and the experiments. One-way ANOVA was also used to determine whether the MC and phenol content of the target cyanobacteria species, when treated with green macroalgae extracts and cell-free filtrate, differed from the control on the last day of the experiment. Data are reported as the means ± standard deviations, where level of significance was *p* < 0.05. Statistical analysis and graphs were created with the program OriginPro, Version 2021 (OriginLab Corporation, Northampton, MA, USA).

## Figures and Tables

**Table 1:** MC-LR (fg cell^−1^) and phenol content (ng cell^−1^) for *Aphanizomenon* sp. BA69 (A), *N. spumigena* BA15 (B), and *Nostoc* sp. BA81 (C) for controls and treatments after the aqueous extract and cell-free filtrate additions obtained from macroalgae *U. intestinalis* after 7 days of the expositions (*n* = 3). Values show arithmetic means and are followed by standard deviations in brackets. Different letters indicate significant differences between the means of the treatments (*p* < 0.05, one-way ANOVA).

Target Cyanobacteria	Control	Extract	Cell-Free Filtrate
	**MC-LR (fg cell^−1^)**		
*Aphanizomenon* sp.	ND	ND	ND
*N. spumigena*	ND	ND	ND
*Nostoc* sp.	92.506 (1.238) ^a^	16.334 (4.288) ^b^	85.125 (4.540) ^a^
	**Phenols (ng cell^−1^)**		
*Aphanizomenon* sp.	0.329 (0.010) ^b^	1.322 (0.016) ^a^	0.125 (0.007) ^c^
*N. spumigena*	1.180 (0.053) ^b^	1.958 (0.027) ^a^	1.887 (0.019) ^a^
*Nostoc* sp.	0.213 (0.004) ^b^	1.237 (0.028) ^a^	0.227 (0.002) ^b^

ND—not detected.

## Data Availability

All data are presented in the article and Supplementary Materials.

## Supplementary Materials

The following are available online at https://www.mdpi.com/article/10.3390/toxins13080589/s1, Figure S1: Phenol concentration (mg L^−1^) of *Aphanizomenon* sp. BA69 (A), *N. spumigena* BA15 (B), and *Nostoc* sp. BA81 (C) for controls (c) and treatments of extract (e), and cell-free filtrate (f) additions obtained from macroalgae *U. intestinalis* after 7 days of the expositions, Table S1: MC-LR (μg L^−1^) and phenol content (mg L^−1^) for *Aphanizomenon* sp. BA69 (A), *N. spumigena* BA15 (B), and *Nostoc* sp. BA81 (C) for controls and treatments after the extract and cell-free filtrate additions obtained from macroalgae *U. intestinalis* after 7 days of the expositions.
