# Supplementary material for: Changes in Growth, Photosynthesis Performance, Pigments, and Toxin Contents of Bloom-Forming Cyanobacteria after Exposure to Macroalgal Allelochemicals
Source: Toxins (Basel). 2021 Aug 23;13(8):589. doi: 10.3390/toxins13080589 (PMC8402365; doi:10.3390/toxins13080589)
Supplement: Supplementary file 1 [file toxins-13-00589-s001.zip › toxins-1300614-supplementary.pdf]

toxins

MDPI

# Supplementary Materials: Changes in growth, Photosynthesis Performance, Pigments, and Toxins Contents of Bloom-Forming Cyanobacteria after Exposure to Macroalgal Allelochemicals

**Gracjana Budzałek, Sylwia Śliwińska-Wilczewska , Marek Klin, Kinga Wiśniewska, Adam Latała and Józef Maria Wiktor**

**Table S1.** MC-LR (μg $L^{-1}$) and phenols content (mg $L^{-1}$) for *Aphanizomenon* sp. CCBA69 (**A**), *N. spumigena* CCBA15 (**B**), and *Nostoc* sp. CCBA81 (**C**) for controls and treatments after the extract and cell-free filtrate additions obtained from macroalgae *U. intestinalis* after 7 days of the expositions.

| Target Cyanobacteria | Control | Extract | Cell-Free Filtrate |
|---|---|---|---|
| | | **MC-LR (μg $L^{-1}$)** | |
| *Aphanizomenon* sp. | ND | ND | ND |
| *N. spumigena* | ND | ND | ND |
| *Nostoc* sp. | 0.204±0.001 | 0.069±0.017 | 0.170 ± 0.010 |
| | | **Phenols (mg $L^{-1}$)** | |
| *Aphanizomenon* sp. | 0.310±0.002 | 5.010 ± 0.020 | 0.089 ± 0.003 |
| *N. spumigena* | 0.804±0.010 | 5.537 ± 0.006 | 0.954 ± 0.006 |
| *Nostoc* sp. | 0.470±0.002 | 5.253 ± 0.015 | 0.454 ± 0.003 |

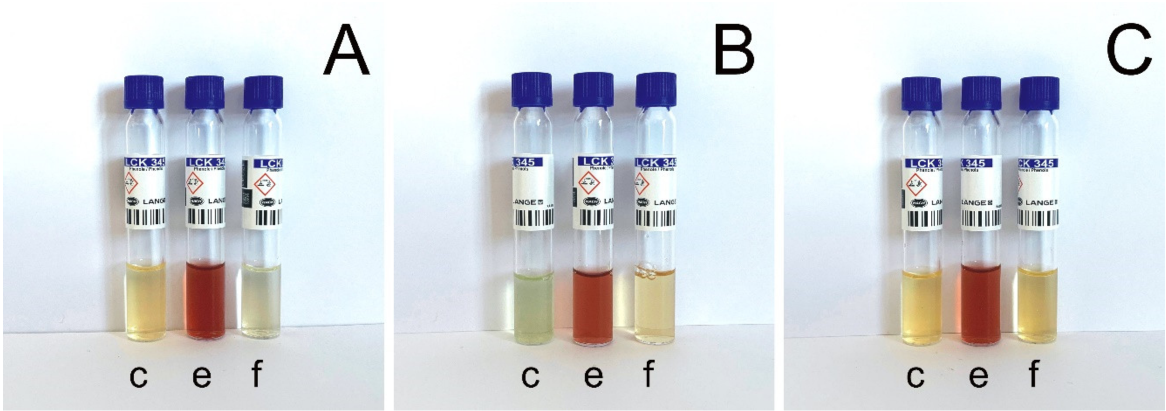


**Figure S1.** Phenols concentration (mg $L^{-1}$) of *Aphanizomenon* sp. CCBA69 (**A**), *N. spumigena* CCBA15 (**B**), and *Nostoc* sp. CCBA81 (**C**) for controls (**c**) and treatments of extract (**e**), and cell-free filtrate (**f**) additions obtained from macroalgae *U. intestinalis* after 7 days of the expositions.
